# Prognostic impact of postoperative radiation in patients undergoing radical esophagectomy for pathologic lymph node positive esophageal cancer

**DOI:** 10.1186/1748-717X-8-116

**Published:** 2013-05-08

**Authors:** Yaping Xu, Jinshi Liu, Xianghui Du, Xiaojiang Sun, Yuanda Zheng, Jianxiang Chen, Bo Li, Wei Liu, Hao Jiang, Weimin Mao

**Affiliations:** 1 Department of Radiation Oncology, Zhejiang Cancer Hospital, Hangzhou, 310022, People’s Republic of China; 2 Department of Thoracic Surgery, Zhejiang Cancer Hospital, Hangzhou, 310022, People’s Republic of China

**Keywords:** Esophageal cancer, Postoperative radiation, Esophagectomy, Survival

## Abstract

**Purpose:**

Though postoperative radiation for esophageal squamous cell carcinoma is offered in selected cases, there is conflicting evidence as to whether it improves overall survival (OS). A retrospective investigation was performed to analyze the prognostic impact of postoperative radiation therapy (PORT) in a large cohort of patients.

**Methods:**

From 2001 to 2009, 725 patients underwent radical esophagectomy (R0) with or without PORT were eligible for retrospective analysis. Patients were grouped into surgery alone (n = 467) and surgery plus PORT (n = 258). Median irradiation doses were 50 Gy (range: 40-56 Gy). Radiation fields encompassed the bilateral supraclavicular fossa, mediastinum, subcarinal area, and the tumor bed for the upper/middle-third disease; the bilateral supraclavicular fossa, mediastinum, the tumor bed, subcarinal area, and lower thoracic paraesophageal area for the lower-third disease. Kaplan-Meier and Cox regression analysis were used to compare OS.

**Results:**

After median follow-up of 53 months, the median OS was 29 months in the PORT group and 23 months in the surgery alone group. The addition of PORT improved OS at 3 years from 36.6 to 43.6% compared with surgery alone. The use of PORT was associated with significantly improved OS (*p* = 0.018). For American Joint Committee on Cancer (AJCC) stage III esophageal cancer (T1-2N2M0, T3N1-2M0, T4N1-3M0), there was significant improvement in OS (*p* = 0.002) in the PORT group, not only for lymph-node metastatic ratio (LNMR) ≥0.25 (*p* = 0.001), but also for LNMR <0.25 (*p* = 0.043). However, for stage IIB disease (T1-2N1M0) there was no significant differences. The addition of POCT didn’t prolong the OS significantly (Surgery alone group, *p* = 0.079; PORT group, *p* = 0.111).

**Conclusions:**

This large retrospective analysis supports the use of PORT for pathologic lymph node positive stage III esophageal squamous cell carcinoma. Given the retrospective nature of this study, the results should be confirmed by appropriately powered randomized trials. Further development of adjuvant therapy in EC is warranted.

## Introduction

Esophageal cancer (EC) is the eighth most common cancer worldwide, and especially in some areas of China is the fourth most common cause of death and is of squamous cell carcinoma (SCC) histology in >90% of cases [1]. For many decades, surgical resection was the only choice for these patients. However, surgery alone has been associated with low cure rates, regardless of surgical approach or histology. Loco-regional recurrence and metastatic spread remain common, despite improvement in surgical techniques and perioperative care. High rates of local and systemic failure have prompted investigation into the multidisciplinary management of those with locally advanced esophageal cancer using neoadjuvant and adjuvant approaches with radiotherapy, chemotherapy, and chemoradiotherapy in an attempt to reduce loco-regional recurrence, and to improve outcome after the surgery. Preoperative radiotherapy and chemotherapy are being used more often, and neoadjuvant chemoradiotherapy is currently the standard of care in many western countries [2-8]. Whether or not postoperative radiotherapy (PORT) and/or postoperative chemotherapy (POCT) affects treatment outcomes, however, remains controversial [9-12].

In this study, a retrospective investigation was performed to 1) identify the postoperative risk factors influencing the outcome following esophagectomy in resectable esophageal squamous cell carcinoma (ESCC) with pathologic lymph nodes positive; 2) propose indications for or against PORT for patients with pathologically positive lymph nodes ESCC after surgery.

## Patients and methods

### Patients’ data

From 2001 to 2009, 1331 patients with thoracic ESCC underwent radical esophagectomy (R0) with or without PORT and/or POCT at a single institution. Patients eligible for the analysis were those with stage T1-4N1-3M0 ESCC. Patients with stage T3-4N0M0 disease were excluded. This was done to remove possible bias, because most patients with T3-4N0M0 disease received PORT which just encompassed the tumor bed. Thus, the comparison was strictly limited to those with stage T1-4N1-3M0 ESCC who received PORT with the regional lymph node and the tumor bed.

Only patients who survived for more than 3 months postsurgery were included in the cohort. This was done to remove possible bias in favor of the PORT group, because some of the patients who received surgery alone might have died in the perioperative period before receiving adjuvant radiation. Thus, the comparison was limited to those who were treated with definitive esophagectomy with or without adjuvant radiation therapy.

Other essential conditions: no distant metastasis, no preoperative chemotherapy and/or radiotherapy, no invasion to cervical esophagus and cardiac part of the stomach.

The present study was approved by the Ethical Review Committee at the hospital (No. zjzlyy-2013-04-87). Recommendations of the Declaration of Helsinki for biomedical research involving human subjects were also followed.

### Staging

Tumor size and extent is coded primarily from the operative report and pathology reports and therefore represents pathologic staging. Extent of nodal disease was determined based on pathologic findings only. This information was used to convert the extent of disease to tumor, node, metastasis staging according to the American Joint Committee on Cancer (AJCC) staging, 7th edition [13]. Table 1 lists the included patients based on their tumor, node, metastasis classification and AJCC stage grouping.

**Table 1:** Patient characteristics based on TNM classification and AJCC stage grouping

	**Stage Grouping**	**PORT**	**Surgery alone**	**Total No. of Patients**
T1-2N1	IIB	20	52	72
T1-2N2	IIIA	23	19	42
T3N1	IIIA	85	167	252
T3N2	IIIB	69	119	188
T4N1-3	IIIC	61	110	171
Total No. of Patients		258	467	725

*TNM,* tumor, node, metastases based classification; *AJCC*, American Joint Committee on Cancer; *PORT*, postoperative radiation therapy.

### Surgery

All patients underwent radical surgery. The standard surgical approach consisted of a limited thoracotomy on the right side and intrathoracic gastric tube reconstruction (Ivor-Lewis procedure) for lesions in the middle/lower-third of the esophagus. Upper-third lesions were treated by neck anastomosis (Mackeown procedure). Most patients underwent two-field lymph node dissection. No pyloroplasty or feeding jejunostomy was performed. A nasogastric tube was placed in each patient until anastomotic sites were closed as assessed by esophagography on post operation day 14.

### Postoperative adjuvant radiotherapy and chemotherapy

In this study, all patients were considered for the PORT and POCT. As the role of PORT and POCT for ESCC was controversial at the time of the treatment of these patients, the utilization of these postoperative adjuvant therapy was thus according to the individual physicians’ preference and the general physical conditions of the patient. Patients with poor prognostic factors such as poor performance status, delayed recovery from surgery, multiple comorbidities, etc did not undergo these adjuvant therapy.

PORT was given to patients with postoperative T1-4N1-3M0 initiated 3 to 4 weeks after the surgery. The extent of the irradiation field was determined based on the primary site in the esophagus. Large T-shaped fields were used that encompassed the tumor bed, bilateral supraclavicular fossa, mediastinum, and subcarinal area for lesions in the upper/middle-third lesions of the esophagus; the tumor bed, bilateral supraclavicular fossa, mediastinum, subcarinal area, and lower thoracic paraesophageal lymph nodes area for lesions in the lower-third lesions of the esophagus. Radiation was given through anteroposterior fields first to 36-40 Gy at 1.8-2 Gy per fraction followed by parallel opposing oblique fields to 10-20 Gy to avoid the spinal cord. Ten MV photons were used to deliver the radiation to the mediastinum through anteroposterior and oblique fields. The bilateral supraclavicular fossas were followed by 9-12 MeV electrons 10-20 Gy. Median irradiation dose was 50 Gy (range: 40-56 Gy). In some cases, targets were shrunk on the basis of the patient’s condition or the physician’s judgment. After 2005, the planning target volume has been irradiated by multiple field arrangement with the use of IMRT techniques. Dose volume histograms for the planning target volume, spinal cord, lung and heart have been calculated in order to gain full knowledge of the 3D dose distribution. The maximum dose to the spinal cord was limited to 45 Gy at any point. The volume of both lungs that received more than 20 Gy (V20) was ≤25% and the heart received 40 Gy (V40) < 50%.

Cisplatin and 5-fluorouracil (≥2 cycles) were used most frequently (70%), although several other chemotherapeutics were also used. Most of patients received sequential chemoradiotherapy. Some patients with good performance status received concurrent chemoradiotherapy.

### Statistical analysis

Overall survival (OS) was determined as the time (in months) from the date of surgery to last follow-up or to the date of death. Survival probabilities were calculated by Kaplan-Meier method and compared by the log-rank test. Multivariate analysis was performed by Cox regression model. Variables in the analysis included gender, age, tumor length, pathologic T-category, pathologic N-category, LNMR, tumor differentiation, PORT, and POCT. Statistical analysis was performed using SPSS software, Version 13.0 (SPSS Inc., Chicago, IL). All probability values were two-sided and *p* values < 0.05 were considered statistically significant.

## Results

A total of 725 patients who underwent radical esophagectomy (R0) were included in the present study: 258 (35.6%) received PORT, 262 (36.1%) received POCT. In 258 PORT patients, 167 (64.7%) received adjuvant chemotherapy, 21 (8.1%) was applied simultaneously. PORT was generally well tolerated. Main toxicity (grade 3 or greater, %): neutropenia 12 (4.7%), thrombocytopenia 5 (1.9%), anaemia 12 (4.7%), nausea/vomiting 11 (4.2%), anorexia 15 (5.8%), dysphagia 30 (11.6%), radiation pneumonitis 17 (6.6%) and fatigue 30 (11.6%). Most side effects were grade I/II and well tolerated by supportive care. The median age of all patients was 56 (range 32–86). Median follow-up period for the surviving patients was 53 months (range 1–97 months). Table 2 lists available patient characteristics and the comparisons by treatment assignment. Patients who received PORT were more often male, < 65 years old and tumor length ≥ 5cm disease.

**Table 2:** Comparison of patient characteristics by treatment assignment (N=725)

**Variable**	**All Patients (%)**	**PORT**		***Pa***
		**Yes**	**No**	
Gender				0.029
Female	68(9)	16(6)	52(11)	
Male	657(91)	242(94)	415(89)	
Age				0.004
< 65	564(78)	216(84)	348(75)	
≥65	161(22)	42(16)	119(25)	
Tumor length				0.012
< 5cm	391(54)	123(48)	268(57)	
≥5cm	334(46)	135(52)	199(43)	
pT-category				0.137
T1-2	123(17)	42(16)	81(17)	
T3	546(75)	198(77)	348(75)	
T4	56(8)	18(7)	38(8)	
pN-category				0.682
N1	351(49)	121(47)	230(49)	
N2	227(31)	86(33)	141(30)	
N3	147(20)	51(20)	96(21)	
LNMR				0.983
< 0.25	582(81)	207(80)	375(80)	
≥0.25	143(19)	51(20)	92(20)	
Tumor differentiation				0.469
High (G1)	90(12)	35(13)	55(12)	
Moderate (G2)	479(66)	176(68)	303(65)	
Low (G3)	156(22)	49(19)	107(23)	
POCT				<0.001
Yes	262(36)	167(65)	95(20)	
No	463(64)	91(35)	372(80)	

^a^*X*^*2*^*p* value; *LNMR*, lymph-node metastatic ratio; *PORT*, postoperative radiation therapy; *POCT*, postoperative chemotherapy.

### Overall survival

**Figure 1:**
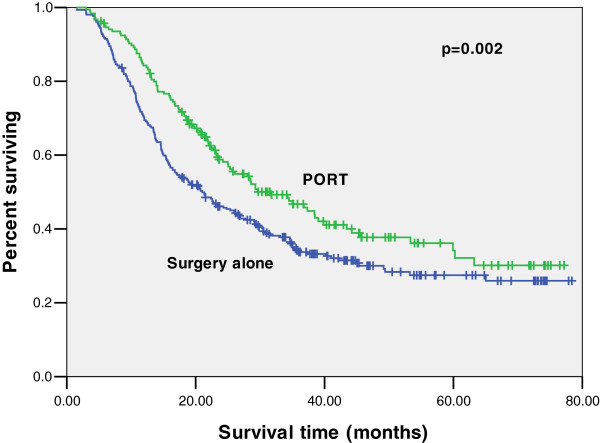
**Kaplan-Meier estimates for overall survival of patients receiving PORT compared with surgery alone for lymph nodes positive stage III esophageal cancer.** The median survival was 29 months for PORT versus 21 months for surgery alone (*p* = 0.002). PORT, postoperative radiation therapy.

The data regarding survival was available for all patients. After median follow-up of 53 months, the median OS was 29 months in the PORT group and 23 months in the surgery alone group. The addition of PORT improved OS at 3 years from 36.6 to 43.8% compared with surgery alone. The use of PORT was associated with significantly improved OS (*p* = 0.018). For American Joint Committee on Cancer (AJCC) stage III esophageal cancer (T1-2N2M0, T3N1-2M0, T4N1-3M0), 414 patients received surgery alone and 238 patients received PORT. Median OS improved from 21 months to 29 months, and 3-year OS improved from 33.7 to 44.9% (*p* = 0.002) (Figure 1). However, for stage IIB disease (T1-2N1M0) there was no significant differences.

### Univariate and multivariate analyses

On unvariate analysis, PORT (hazard ratio [HR] 0.79, 95% confidence interval [CI] 0.65 – 0.97, *p* = 0.018) was associated with improved survival. POCT did not significantly improve OS. Male, ≥65 years old, higher T category, more lymph nodes metastases and higher LNMR were all associated with decreased OS. On multivariate analysis, use of PORT was again associated with improved survival (HR 0.77, 95% CI 0.63 – 0.94, *p* = 0.001). Male gender, higher T stage and more lymph nodes metastases were again associated with decreased survival (Table 3).

**Table 3:** Univariate and mutivariate analysis for survival

**Variable**	**Univariate analysis**			**Mutivariate analysis**		
	**CHR**	**95% CI**	***P***	**CHR**	**95% CI**	***P***
Gender						
Female	1			1		
Male	0.70	1.18-2.44	0.004	1.44	1.00-2.08	0.049
Age						
< 65	1			1		
≥65	1.26	1.02-1.56	0.031	1.17	0.93-1.46	0.175
Tumor length						
< 5cm	1			1		
≥5cm	1.01	0.84-1.22	0.897	0.95	0.79-1.15	0.618
pT-category						
T1-2	1			1		
T3	1.42	1.09-1.87	0.011	1.48	1.12-1.95	0.005
T4	2.65	1.82-3.87	< 0.001	2.27	1.53-3.35	<0.001
pN-category						
N1	1			1		
N2	1.39	1.12-1.74	0.003	1.33	1.06-1.67	0.015
N3	2.70	2.14-3.40	< 0.001	1.79	1.29-2.48	< 0.001
LNMR						
< 0.25	1			1		
≥0.25	2.36	1.91-2.91	< 0.001	1.54	1.14-2.07	0.004
Tumor differentiation						
High (G1)	1			1		
Moderate (G2)	0.88	0.66-1.17	0.383	0.88	0.67-1.19	0.423
Low (G3)	1.13	0.82-1.56	0.463	0.94	0.67-1.30	0.689
PORT						
No	1			1		
Yes	0.79	0.65-0.97	0.018	0.77	0.63-0.94	0.001
POCT						
No	1			1		
Yes	1.10	0.91-1.33	0.325	1.15	0.91-1.44	0.061

*CHR*, Cox hazard ratio; 95% *CI*, 95% Confidence Interval; *LNMR*, lymph-node metastatic ratio; *PORT*, postoperative radiation therapy; *POCT*, postoperative chemotherapy.

### Overall survival by metastatic lymph-node ratio

**Figure 2:**
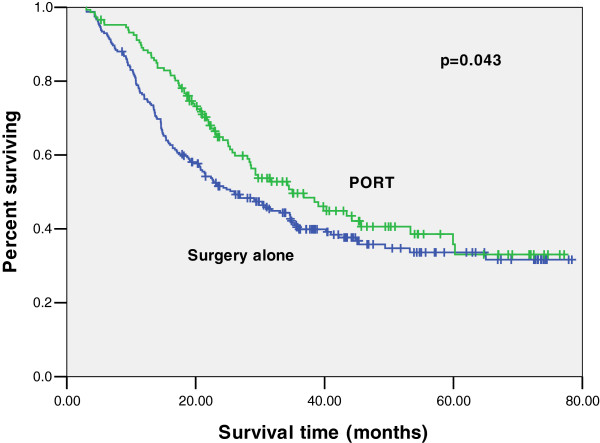
**Kaplan-Meier estimates for overall survival of patients receiving PORT compared with surgery alone for lymph nodes positive stage III esophageal cancer with LNMR < 0.25.** The median survival was 35 months for PORT versus 29 months for surgery alone (*p* = 0.043). PORT, postoperative radiation therapy; LNMR, lymph-node metastasis ratio.

**Figure 3:**
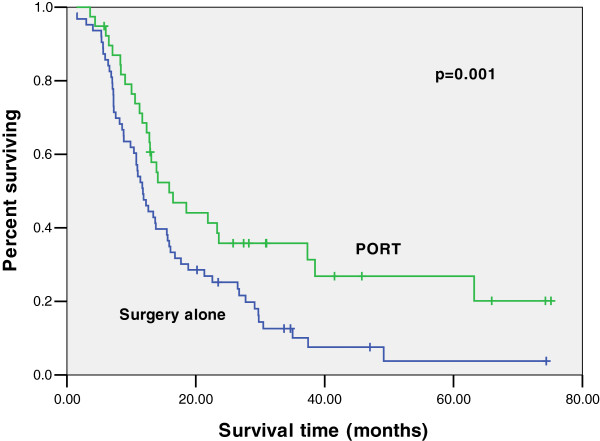
**Kaplan-Meier estimates for overall survival of patients receiving PORT compared with surgery alone for lymph nodes positive stage III esophageal cancer with LNMR >0.25.** The median survival was 18 months for PORT versus 11 months for surgery alone (*p* = 0.001). PORT, postoperative radiation therapy; LNMR, lymph-node metastasis ratio.

A total of 375 patients with LNMR < 0.25 received surgery alone, compared with 207 patients who received PORT. Median OS was improved from 30 months to 34 months with the addition of PORT as well as an improvement in 3-year OS from 43.7 to 46.2%, but there was no significant difference (*p* = 0.191). When these patients were grouped by AJCC stage, there was no OS benefit for IIB disease (*p* = 0.062). For stage III LNMR < 0.25, 323 patients received surgery alone and 188 patients received PORT. Median OS improved from 29 months to 35 months, and 3-year OS improved from 41.1 to 47.9% (*p* = 0.043) (Figure 2). Similarly, when analyzing patients with LNMR ≥0.25, there was no significant difference for stage IIB disease (*p* = 0.317). However, for stage III disease, median OS improved from 11 months to 18 months, and 3-year OS improved from 9.2 to 24.5% (*p* = 0.001) (Figure 3).

### Overall survival by combining chemotherapy

A total of 167 patients received PORT combined with POCT, compared with 91 patients who received PORT alone. The median OS and 3-year OS rate were 37 months and 47.6% with PORT alone, 24 months and 40.4% with PORT plus POCT. When these patients were treated with concurrent radiochemotherapy, median OS was 26 months. The addition of chemotherapy didn’t prolong the OS (*p* = 0.111) in PORT group. Similarly, when analyzing patients with surgery alone group, the median OS and 3-year OS rate were 25 months and 38.0% with surgery alone, 14 months and 30.6% with surgery plus POCT (*p* = 0.079).

## Discussion

The results of this large retrospective study revealed that the addition of PORT is associated with significantly improved OS for AJCC lymph node positive stage III ESCC. When stratifying by the LNMR, the survival benefit associated with postoperative remains for stage III disease. For stage IIB (T1-2N1M0) patients, there was no significant OS benefit with the use of PORT. However, in our study the number of stage IIB patients was only 72; the limited number of events made it difficult to evaluate OS differences for this stage disease. Other positive prognostic factors for overall survival include gender, pathologic T classification and N classification.

In spite of radical resection and extended lymph node dissection, most esophageal cancer patients still die of recurrence. Although there is no general recommendation for adjuvant radiotherapy, it has been shown that prophylactic radiotherapy after radical operation of esophageal cancer can reduce the local recurrence rate [14]. In an attempt to further clarify the impact of PORT in a prospective fashion, several randomized studies were performed comparing PORT with surgery alone [11,12,15-17]. However, the majority of the evidence has revealed that PORT does not confer any survival benefit over surgery alone. Teniere et al. evaluated 221 patients with epidermoid carcinoma of the middle to lower third of the esophagus who were randomized to PORT to a dose of 45–55 Gy versus observation. They found that although local control improved from 15 to 30%, there was no survival benefit with the addition of PORT [15]. Zieren et al. randomized 68 patients with squamous cell carcinoma to either PORT or surgery alone and found that PORT significantly increased the fibrotic stricture rate and did not improve OS or disease free survival [12]. Therefore, there are little data to suggest that PORT affords any survival benefit [9]. However, all of the previous mentioned trials did not stratify the patients based on their stage and likely were not large enough to detect an improvement in survival only for those patients with lymph nodes positive or deeply invading tumor. In addition, both Teniere et al. [15] and Zieren et al. [12] included patients with positive celiac nodes (stage M1). These patients are excluded from our study and represent a cohort at much higher risk for distant failure and therefore are less likely to benefit from PORT therapy. Adjuvant radiotherapy can theoretically treat microscopic disease left behind after surgery and increase local control.

Recently, Schreiber et al. performed a retrospective review using the Surveillance Epidemiology and End Results (SEER) database to analyze whether there was survival benefit to adjuvant radiation in stage T3-4N0M0 or T1-4N1M0 esophageal cancer who were definitively treated with esophagectomy. A total of 1046 patients met the selection criteria: 683 (65.3%) received surgery alone and 363 (34.7%) received PORT. For AJCC stage III esophageal carcinoma (T3N1M0 or T4N0-1M0), 346 patients underwent surgery alone and 231 patients received PORT. Use of PORT resulted in an improvement in median OS from 15 months to 19 months and an improvement in 3-year OS from 18.2 to 28.9% (*p* < 0.001), respectively. This benefit was present for both squamous cell carcinoma and adenocarcinoma [10]. Other studies have also addressed the impact of PORT on esophageal squamous cell carcinoma with lymph nodes positive, which found a survival benefit only for those with stage III patients [11,18]. Similar to the findings mentioned above, our study revealed that PORT significantly improved OS for patients with lymph node positive stage III disease, and suggested that it is essential to use proper patient selection criteria when the decision-making for postoperative adjuvant radiotherapy in ESCC.

The role of adjuvant chemotherapy of ESCC has been addressed in a number of clinical phase III trials [19-21]. Ando et al. conducted a randomized multicenter trial to determine whether POCT improves the outcome of patients with ESCC who underwent radical surgery. Their results showed that the 5-year overall survival rate was 52% with surgery alone, and 61% with surgery plus chemotherapy (one-sided log-rank, *p* = 0.13). Risk reduction by POCT was remarkable in the subgroup with lymph node metastasis [19]. Zhang et al. conducted a meta-analysis comprising a total of 1000 patients with esophageal cancer (ESCC and adenocarcinoma) in 2008. The patients with pathologically positive lymph nodes demonstrated a positive trend towards improved survival, but this was not significant (OR, 0.76; 95% CI: 0.538-1.083) [22]. The theoretical advantages of adding adjuvant chemotherapy to the treatment of esophageal cancer are for potential targeting micrometastatic disease, thus decreasing the risk of distant spread. Nevertheless, our study showed that the therapeutic efficacy of POCT was unsatisfactory. This might be attributed to the fact that our study was retrospective and the chemotherapy regimens were not quite the same among these patients. Therefore, the therapeutic efficacy of POCT in these patients is still no clear indication based on our data. Recently, in a phase II non-randomized trial which evaluated postoperative concurrent chemoradiation with cisplatin and 5-fluorouracil in patients with poor prognosis oesophageal and gastroesophageal junction (EGJ) cancers, the 4-year overall survival, freedom from recurrence, distant metastatic control and locoregional control were 51%, 50%, 56% and 86% respectively for patients with lymph node positive (T3 or T4) tumours, which are better than the historical outcomes with surgery alone [23]. However, the efficacy of postoperative chemoradiation has not been compared to surgery alone in a randomized trial in patients with EC. Therefore, evaluation of postoperative chemoradiotherapy for these patients may be important. Further development of postoperative adjuvant therapy in EC is warranted.

In addition, the use of neoadjuvant chemoradiotherapy has become an increasingly used treatment approach. The potential value of preoperative therapy is that adjuvant therapy could be started immediately targeting any micro metastatic deposits without allowing time for further growth, and treatment would not be given until diagnosis and staging is firmly assessed. In addition, prior to surgery it is thought that the patient’s may be better able to tolerate aggressive chemotherapy and radiation as it can start immediately and their physical and nutritional state has not been burden by the need to recover from surgery. Results from a recent multicenter phase III randomized trial (CROSS study) showed that neoadjuvant chemoradiotherapy improved OS compared to surgery alone in patients with resectable (T2-3N0-1M0) esophageal or EGJ cancers. Median survival was 49 months in the neoadjuvant chemoradiotherapy arm compared to 26 months in the surgery alone arm [23]. In 2011, Kranzfelder et al. published a meta-analysis which sought to clarify the benefits of neoadjuvant treatment for ESCC. Nine RCTs involving neoadjuvant chemoradiotherapy versus surgery, eight involving neoadjuvant chemotherapy versus surgery, and three involving neoadjuvant treatment followed by surgery or surgery alone versus definitive chemoradiotherapy were identified. The HR for overall survival was 0.81 (95% CI: 0.70-0.95; *p* = 0.008) after neoadjuvant chemoradiotherapy and 0.93 (0.81-1.08; *p* = 0.368) after neoadjuvant chemotherapy. The likelihood of R0 resection was significantly higher after neoadjuvant treatment (Chemoraditherapy: HR 1.15, p = 0.043; chemotherapy: HR 1.16, p = 0.006). Morbidity rates were not increased after neoadjuvant Chemoradiotherapy (HR 0.94, p = 0.363) but 30-day mortality was non-significantly higher with combined treatment. Morbidity (HR 1.03, p = 0.638) and mortality (HR 1.04, p = 0.810) rates after neoadjuvant chemotherapy and surgery did not differ from those after surgery alone. The authors concluded that the patients with resectable ESCC, a significant survival benefit for neoadjuvant chemoradiotherapy was evident, with no increase in morbidity rate [24]. There are not well done randomized trials to compare the outcome of postoperative therapy against preoperative therapy in esophageal cancer with modern staging and modern treatment techniques. Further development of the multidisciplinary management for patients with locally advanced esophageal cancer after surgery using adjuvant treatment compared to neoadjuvant chemoradiotherapy is warranted. The approach is currently being explored in China by investigators of the ZTOG1201 trial, a multicenter phase II trial of neoadjuvant and adjuvant chemoradiotherapy in locally advanced EC (NCT01463501)[25].

In conclusion, for those who do undergo primary surgery, the results of this large population-based analysis revealed that there is an association of improved OS with PORT for lymph nodes positive stage III EC. Our results suggest that a subset of such patients may benefit from aggressive local therapy. Given the retrospective nature of this study, until appropriately powered randomized trials confirm these results, caution should be used before broadly applying these findings in clinical practice. As a retrospective study, our results do not have the same strength as a prospective study, however, it provides a basis for the design of future randomized, prospective clinical trials.

## Competing interests

The authors declare that they have no competing interests.

## Authors’ contributions

YX, JL and XD contributed equally in analysis of data and drafting the manuscript; XS, YZ, JC, BL, WL and HJ collected the data and provided the critical revision of the manuscript; WM provided the conception of this study and the final approval of the version to be published. And all authors read and approved the final manuscript.
